# Preparation and Performance of Lignin-Based Multifunctional Superhydrophobic Coating

**DOI:** 10.3390/molecules27041440

**Published:** 2022-02-21

**Authors:** Xue Liu, Chao Gao, Chenglong Fu, Yuebin Xi, Pedram Fatehi, Joe R. Zhao, Shoujuan Wang, Magdi E. Gibril, Fangong Kong

**Affiliations:** 1 State Key Laboratory of Biobased Material and Green Papermaking, Qilu University of Technology, Shandong Academy of Sciences, Jinan 250353, China; 15838118014@163.com (X.L.); 15864029637@163.com (C.G.); fcl1z66@163.com (C.F.); qlgdxyb@qlu.edu.cn (Y.X.); 2 Chemical Engineering Department, Lakehead University, 955 Oliver Road, Thunder Bay, ON P7B 5E1, Canada; pfatehi@lakeheadu.ca; 3 Tri-Y Environmental Research Institute, Vancouver, BC V5M 3H9, Canada; joezhao228@yahoo.ca

**Keywords:** superhydrophobic, lignin particles, mechanical durability, chemical stability, self-cleaning

## Abstract

Superhydrophobic coatings have drawn much attention in recent years for their widespread potential applications. However, there are challenges to find a simple and cost-effective approach to prepare superhydrophobic materials and coatings using natural polymer. Herein, we prepared a kraft lignin-based superhydrophobic powder via modifying kraft lignin through 1H, 1H, 2H, 2H-perfluorodecyl-triethoxysilane (PFDTES) substitution reaction, and constructed superhydrophobic coatings by direct spraying the suspended PFDTES-Lignin powder on different substrates, including glass, wood, metal and paper. The prepared lignin-based coatings have excellent repellency to water, with a water contact angle of 164.7°, as well as good friction resistance, acid resistance, alkali resistance, salt resistance properties and quite good self-cleaning performance. After 30 cycles of sand friction or being stayed in 2 mol/L HCl, 0.25 mol/L NaOH and 2 mol/L NaCl solution for 30 min, the coatings still retain super hydrophobic capability, with contact angles higher than 150°. The superhydrophobic performance of PFDTES-Lignin coatings is mainly attributed to the constructed high surface roughness and the low surface energy afforded by modified lignin. This lignin-based polymer coating is low-cost, scalable, and has huge potential application in different fields, providing a simple way for the value-added utilization of kraft lignin.

## 1. Introduction

With the development of science and technology, people’s consumption of energy, especially fossil based chemicals, is faster and faster, resulting in the scarcity of resources on the earth, so it is urgent to seek renewable energy sources. Among them, biomass has attracted social attention due to its renewable and abundant reserves [1,2]. Lignin, the second most abundant biomass after cellulose and the only aromatic biopolymer in plants, has extensively been sought for producing numerous functional materials [3]. Zhang et al. prepared a multifunctional hydrogels with super toughness using sodium lignosulfonate (LS) and biodegradable poly (vinyl alcohol) (PVA) as raw materials [4]. Liu et al. successfully synthesized removable and strong bio-based polyurea adhesives via substituting polyetheramine partially with polyetheramine-grafted lignin, introducing a chain extender containing dynamic disulfide bonds. Moreno et al. synthesized tough and transparent nanocomposites via Pickering emulsion polymerization using biocatalytic hybrid lignin nanoparticles [5]. Lignin has gradually been developed to an excellent candidate material for biomass modification and functionalization [6].

Superhydrophobic materials are widely used in biomedical, environmental engineering, new energy materials and other fields due to their anti-icing [7,8,9], anti-corrosion [10], self-cleaning [11], oil-water separation [12,13], etc. However, most of the superhydrophobic materials reported at present are based on petroleum materials, which cause great pressure on environmental protection. Therefore, biomass-based superhydrophobic materials are being paid more and more attention in recent years.

Lignin, as a cost-effective, environmentally friendly and renewable natural polymer, has benzene ring rigid structure [14,15], which makes that lignin become an attractive material for the synthesis of biomass superhydrophobic materials [16]. Research has found that there are two essential factors for constructing superhydrophobic surfaces, one is sufficiently low surface energy, and the other is micro-nano composite structure with high surface roughness [17,18,19]. However, lignin, containing hydrophilic groups such as hydroxyl groups and carboxyl groups, does not have low surface energy and high surface roughness due to the lack of regular micron-level fluctuation on lignin surface [20]. Therefore, it is necessary to modify lignin appropriately and construct micro-nano composite structure to meet the conditions of producing superhydrophobic surface with lignin. Yu et al. produced a micrometer-sized porous oil/water separation material with superhydrophobic surface via one-step synthesis using kraft lignin [21]. Wang et al. [22] presented a simple method for the fabrication of covalent-noncovalent forces stabilizing lignin nanospheres (HT-LNS), which can be employed in the preparation of superhydrophobic coatings. Oribayo et al. [23] reported the synthesis of lignin-based polyurethane (LPU) foam for utilization in spill clean-ups. In current research on lignin-baesd superhydrophobic materials, there are still a series of problems such as complicated processes, insufficient hydrophobicity, low graft rate and so on. Therefore, it is necessary to further seek a simple process for preparing lignin-based materials with high superhydrophobility.

In this work, we prepared a novel lignin-based micro/nano structure coating with superhydrophobic property by a facile and inexpensive spray method. The contact angle for water on this coating is greater than 150°. First, the kraft lignin was modified by PFDTES through replacing hydrophilic groups of lignin with PFDTES to form PFDTES-Lignin. After being sprayed on various substrates, the PFDTES-Lignin coating surfaces exerts excellent hydrophobicity with contact angle as high as 164.7°. The coating not only owns the characteristics of easy biodegradation and renewability, but also has good mechanical properties, chemical stability, self-cleaning performance, illustrating a huge application potential on the surface of different substrates.

## 2. Results and Discussion

**Figure 1:**
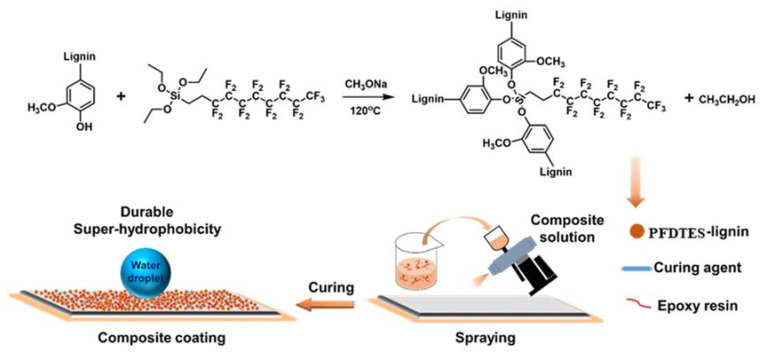
Schematic illustration of lignin modification and the durable superhydrophobic coating fabrication process.

The scheme of the fabrication process of kraft lignin is presented in Figure 1. The superhydrophobic PFDTES-Lignin particles were obtained by fluorination modification of kraft lignin. The lignin-based superhydrophobic coating was prepared by spraying -PFDTES-Lignin on various substrates.

### 2.1. Lignin Modification and Characterization

**Figure 2:**
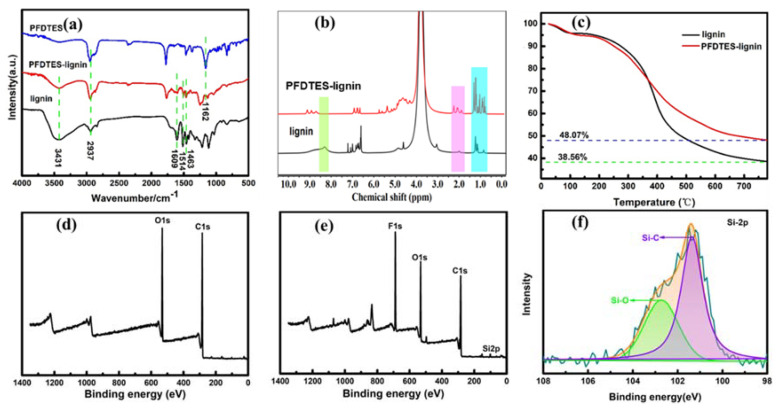
Characterization of modified lignin (**a**) FTIR spectra of lignin, PFDTES-Lignin and PFDTES, (**b**) 1H NMR spectra of lignin and PFDTES-Lignin, (**c**) Weight loss of lignin and PFDTES-Lignin, (**d**,**e**) The XPS spectra of lignin and PFDTES-Lignin. (**f**) Si-2p XPS spectra obtained from PFDTES-Lignin sample.

FTIR spectra of lignin and PFDTES-Lignin are presented in Figure 2a. Both lignin and PFDTES-Lignin have an absorption peak at 3431 cm^−1^, which is caused by the stretching vibration of hydroxyl groups in aromatic and aliphatic structure [24]. Three strong characteristic absorption peaks at 1609, 1514, and 1463 cm^−1^ affirm the presence of aromatic skeletal structure from lignin and modified-lignin [25]. The typical absorption peak at 2937 cm^−1^ belongs to the C-H stretching vibration of lignin, PFDTES-Lignin and PFDTES. The presence of absorption peak at 1162 cm^−1^, stretching vibration of the C-F bond in PFDTES, in the spectrum of PFDTES-Lignin [26] indicates that PFDTES were successfully grafted onto lignin.

The surface component analysis of lignin and PFDTES-Lignin were examined using X-ray photoelectron spectroscopy (XPS). The results show that F content is 19.12%, corresponding to 36.1% graft ratio of PFDTES. Figure 2d shows that lignin is mainly composed of O (532 eV) and C (284 eV). PFDTES-Lignin owns elements F (689 eV) and Si (152.9 eV, 101 eV) except element O and element C (Figure 2e). The Si 2p peaks in the PFDTES-Lignin spectrum can be curve-fitted to two peaks at binding energies of 102.76 eV and 101.34 eV (Figure 2f), which corresponds to Si-O and Si-C respectively [27,28,29]. The Si-O is the combination of silicon and oxygen key on the benzene ring of lignin, which can be supported by phenolic hydroxyl groups test of lignin and PFDTES-Lignin. The phenolic hydroxyl content of lignin and PFDTES-Lignin are 3.85 mmol/g and 2.53 mmol/g respectively, which can be used to calculate the reaction ratio of PFDTES, 34.3%, which is similar to 36.1% of the XPS result. The ^1^H NMR data in Figure 2b also showed the similar results. It can be observed that the phenolic hydroxyl signal intensity of lignin at 8–8.5 ppm [30] was reduced in the PFDTES-Lignin. Moreover, due to the introduction of PFDTES in the modified lignin, the signal strength at 0.7–1.3 ppm (methyl) and 1.5–1.8 ppm (methylene) are much stronger than that of lignin [31], which further demonstrates the successful graft of PFDTES onto lignin.

At the same time, the thermal stability of lignin and PFDTES-Lignin was investigated. The results are presented in Figure 2c. Before the temperature reaches 150°, lignin and PFDTES-Lignin have a slight weight loss, which is mainly caused by the loss of water in the sample [32]. In the temperature range of 200 °C to 600 °C, lignin has a large weight loss, which is mainly due to the thermal decomposition of lignin, that is, the break of carbon-carbon bond between lignin units and the fatty side chain of aromatic ring [33,34]. The weight loss of PFDTES-Lignin is much lower than that of lignin at 200–400 °C, which is mainly due to the graft of perfluorocarbon triethoxysilane onto lignin [35,36,37], while in the range of 400–600 °C lignin and PFDTES-Lignin both give a similar decomposition trend. The final residual amount of PFDTES-Lignin, 48.07%, was higher than that of original lignin, 38.56%, which is a result of the existence of inorganic group Si-O bond on the surface of PFDTES [38].

### 2.2. Characterization of PFDTES-Lignin Coating

**Figure 3:**
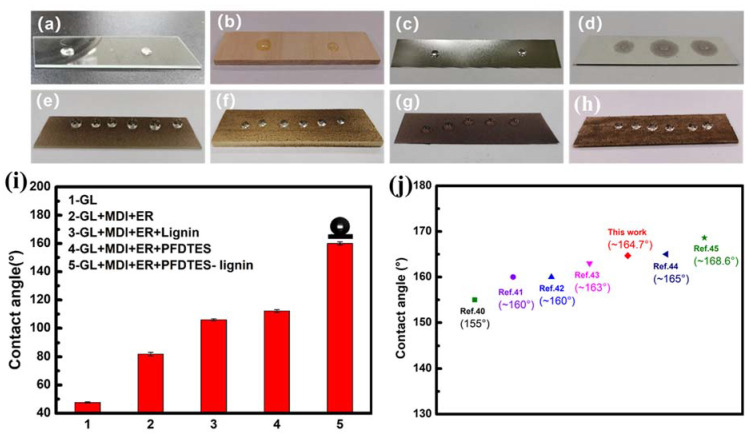
The wettability of water droplets on the surface of uncoated glass (**a**), wood sheet (**b**), metal sheet (**c**), paper (**d**) and the surface of Lignin-PFDTES coated glass (**e**), Lignin-PFDTES coated wood sheet (**f**), Lignin-PFDTES coated metal sheet (**g**) and Lignin-PFDTES coated paper (**h**). (**i**) Comparison of contact angles of different coatings on glass substrate. (**j**) Comparison of contact angles of our sample with samples reported in the literatures.

Automatic contact angle measurement instrument (OCA50 Dataphysics, Germany) was used to measure the static contact angle (CA) between the water droplets and the surfaces of the coatings. In order to ensure the rigor of the experiment, the suspension method was used at room temperature. The selected liquid was deionized water, and the volume of water droplets was 4 μL. Figure 3a–h showed the comparison of wettability between four original substrates and PFDTES-Lignin coated substrates. All the contact angles of the original substrates were less than 90°, which was hydrophilic, and the water on wood chips and paper was even directly absorbed, while the PFDTES-Lignin coating on glass had a high contact angle of up to 164.7°. Figure 3i is a comparison of contact angles of different coatings on glass substrate. It’s worth noting that the glass slides coated with curing agent and epoxy resin, glass slides coated with curing agent, epoxy resin and lignin, glass slides coated with curing agent, epoxy resin and PFDTES all didn’t give a superhydrophobic performance. This is because curing agent as well as epoxy resin do not have low surface energy and mastoid structure with micro-nano roughness. Also, lignin itself does not have low enough surface energy. Among the chemicals used in this work, PFDTES has relatively low surface energy due to fluorine, while it does not have mastoid structure with micro-nano roughness. As reported in the literature [39], low surface energy and micro-nano roughness are important factors to achieve super hydrophobicity. Compared with the superhydrophobicity of sawdust modified products [40], lignin-modified hydrophobic coatings [41,42,43] and coatings of other materials [44,45] reported in the literatures, our sample, as shown in Figure 3j, has a hydrophobicity of 164.7°, occupying an advantage among superhydrophobic modified products.

**Figure 4:**
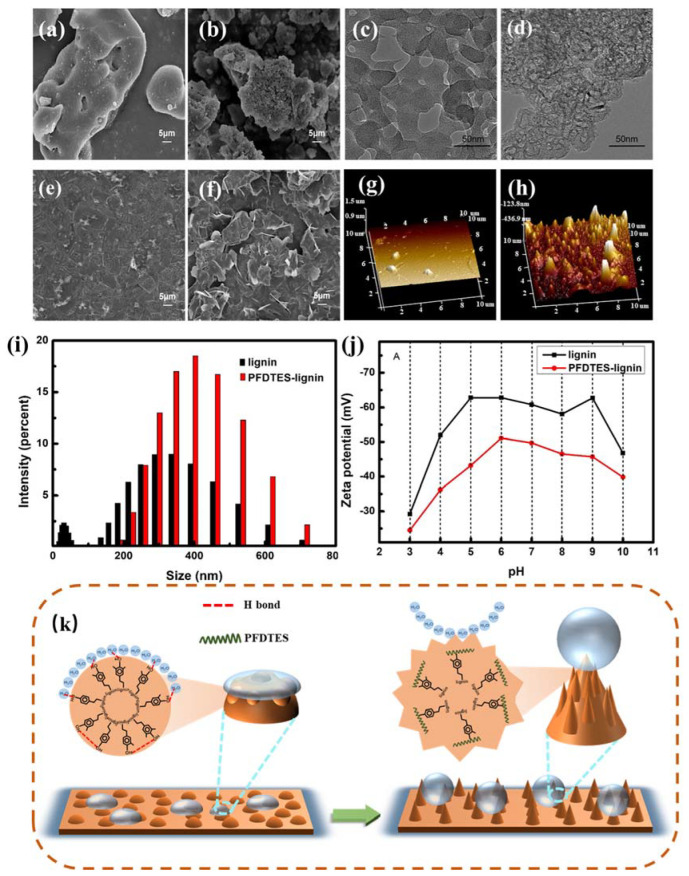
Surface morphology of PFDTES-Lignin coating substrate. (**a**,**b**) The SEM images of original lignin and modified lignin particles. (**c**,**d**) The TEM images of original lignin and modified lignin particles. (**e**,**f**) SEM images of unmodified lignin and modified lignin coating, respectively, (**g**,**h**) AFM images with original lignin and modified lignin coating, respectively, (**i**) DLS distribution of lignin and PFDTES-Lignin, (**j**) Zeta potential of lignin and PFDTES-Lignin samples at different pH values. (**k**) Schematic diagram of mechanism.

In order to exploring the mechanism of good hydrophobicity of PFDTES-Lignin, we observed the surface microstructure of lignin coating and PFDTES-Lignin coating using SEM and AFM. As presented in Figure 4e,f, the SEM diagram showed that lignin coating surface was relatively smooth, while PFDTES-Lignin coating surface was very rough. Similarly, from the AFM image (Figure 4h), it can be found that PFDTES-Lignin coating was significantly rougher than lignin coating (Figure 4g), and the corresponding root-mean-square surface roughness (Rq) is 56 nm. The above phenomenon was related to the fact that lignin is soluble in acetone and PFDTES-Lignin is insoluble and suspended in acetone.

We further observed the SEM and TEM of lignin and PFDTES-Lignin. As shown in Figure 4a,b, the surface of the original lignin is very smooth, while the surface of the PFDTES-Lignin particles become very rough, similar to the surface of weathered rock covered with small papillae. TEM images (Figure 4c,d) showed the same results that protolignin was elliptic, whereas PFDTES-Lignin particles resembled polygons. The whole piece of lignin became loose after modification. We hypothesized that this is because the hydrogen bond in the molecule is broken after the phenolic hydroxyl groups on the lignin are replaced by perfluorosilane, then the distance between molecules is increased. To verify our conjecture, the particle size and Zeta potential of lignin and PFDTES-Lignin were analyzed. Figure 4i shows that the particle size of PFDTES-Lignin is mainly distributed in the range of 260~600 nm, which is larger than that of lignin whose particle size mainly distributed in the range of 30~500 nm, certainly, the average particle size of PFDTES-Lignin (600 nm) is larger than that of lignin (390 nm). Figure 4j shows that the electrification of PFDTES-Lignin is lower than that of lignin in the pH range of 3 to 10, which is attributed to the replacement of the charged group -OH by perfluorosilane which afforded the PFDTES-Lignin with low surface energy [46]. The internal chains of lignin molecules were pulled apart, resulting in the roughness of lignin surface, which creates the necessary conditions for the micro-nano roughness, which is required for the post-production of super hydrophobic coatings. A corresponding mechanism related with the hydrophobicity is provided in Figure 4k, where we can conclude that the change of hydrophobicity before and after lignin modification is related to the transformation of coating surface from Wenzel state to Cassie-Baxter state. This is due to that with the modification of lignin, PFDTES replaces the active hydroxyl group on lignin, which reduces its surface energy and weakens the attraction between chains in lignin molecules, then the entangled chains of lignin molecules are separated, making the surface of PFDTES-Lignin becomes very rough. In conclusion, the two important factors of superhydrophobic construction are micro-nano roughness and low surface energy [47].

**Figure 5:**
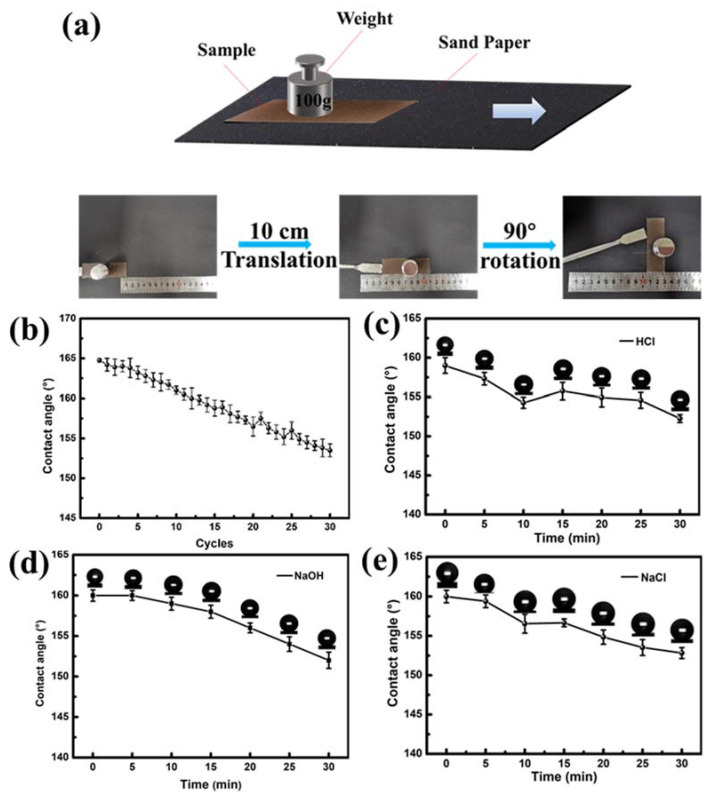
Mechanical properties and chemical durability of PFDTES-Lignin superhydrophobic coatings. (**a**) Sanding experiment process, (**b**) Contact angles of the sample sanding experiment under different cycles, (**c**) Acid resistance of superhydrophobic coatings, (**d**) Salt tolerance of superhydrophobic coatings, (**e**) Alkali resistance of superhydrophobic coatings.

Good mechanical properties and chemical durability under adverse conditions are important indexes for the superhydrophobic application of modified lignin. In this experiment, the mechanical properties of the coating were tested using a sanding test. For the sandpaper friction test, the coating with a weight of 100 g on its upside was placed face-down to the 800 mesh sandpaper (0.5 kPa). We defined the process that the sample was moved for 10 cm along the ruler, and then rotated by 90° as one cycle of the sandpaper abrasion test. The sanding test is illustrated in Figure 5a. Then the static water contact angles were surveyed after each abrasion cycle [47]. The superhydrophobic coating was immersed in 2 mol/L HCl, 0.25 mol/L NaOH and 2 mol/L NaCl solution for some time to investigate its chemical stability. The results (Figure 5b) show that after 30 cycles of sanding, the contact angle of the coating is still as high as 150°. Figure 5c,e shows that compared with the unsoaked coating, although the contact angle decreased after immersion in HCl and NaCl solution for 30 min, the coating still retained super repellency to water. Furthermore, it can be seen in Figure 5c, PFDTES-Lignin coating also has certain alkali resistance and all the contact angles are greater than 150°, which indicates that PFDTES-Lignin superhydrophobic coating can be used under harsh environmental conditions.

**Figure 6:**
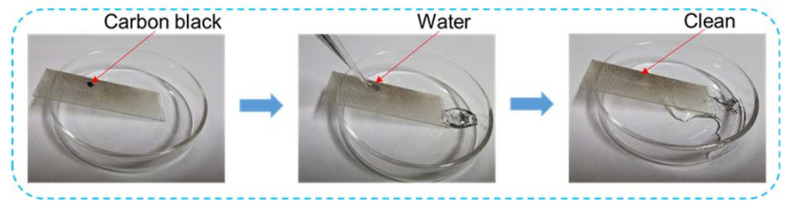
Testing process of self-cleaning performance of PFDTES-Lignin coating.

The coating surface attached to the substrate is easily exposed to dirt, oil stains and other dirty environments. The pollutants may hide in the gaps of the micro-nano structure, thus destroy the micro-structure of the superhydrophobic coating surface and further lead to the loss of the super-hydrophobic performance. Therefore, the coating must have self-cleaning property. In this experiment, carbon black was used as an artificial pollutant, and it was sprinkled on the surface of PFDTES-Lignin coating on glass. Then, water drops were applied on the surface of the coating with a dropper to test the self-cleaning performance of the coating. As shown in Figure 6, water droplets roll down from the coating and wash down the carbon black on the surface of the coating, which fully proves that the PFDTES-Lignin coating has self-cleaning and anti-fouling properties, which make it a broader application in such industries as furniture supplies and catering.

## 3. Materials and Methods

### 3.1. Materials

The kraft lignin was produced by acidification of black liquor from kraft pulping process, and it was purified by the alkali-acid treatment as follows. First, the lignin was dissolved with 0.5 mol/L NaOH solution and the insoluble matter was removed by centrifugation, then the lignin was precipitated by adjusting the solution pH to 2.0 with 0.5 mol/L sulfuric acid solution. After centrifugation, the precipitate was washed to neutral and dried in an oven at 60 °C. Sodium methoxide, 1*H*, 1*H*, 2*H*, 2*H*-perfluorodecyltriethoxysilane (PFDTES) and *N*,*N*-dimethylformamide (DMF, 99.5%) were purchased from Shanghai Alighting Reagent Co., Ltd. (Shanghai, China). Ethanol (99.7%), acetone (99.5%), E-44 epoxy resin and 4′4-Methylene bis were purchased from Shanghai McLean Biochemical Technology Co., Ltd. (Shanghai, China). All reagents were used without further purification.

### 3.2. Preparation of PFDTES-Lignin

The PFDTES-Lignin were prepared by grafting reaction. 3 g of kraft lignin was dissolved in 30 mL of DMF solution, and the solution was treated by an ultrasound for 20 min. 0.084 g of sodium methanol as initiator was dissolved in 4 mL methanol solution. The above two solutions were then poured into a 150 mL of four-neck flask and mixed well. Then, the mixture was heated to 120 °C firstly and then PFDTES as low surface energy component was added into the solution and stirred for 4 h. When the reaction was finished, the dark brown sediment was collected by centrifugation, and washed with 500 mL ethanol for three times to remove the unreacted substances, and dried using freeze drying.

### 3.3. Fabrication of Superhydrophobic Coatings on Various Substrate

The PFDTES-Lignin was applicable to various substrates. Superhydrophobic lignin coatings are prepared using four substrates including glass, metal sheet, wood, and coated paper. The specific method was as follows, 1.5 g of epoxy resin as a crosslinking agent was added in 15 mL of acetone and stirred for 10 min, then 0.8 g of PFDTES-Lignin and 1.5 g of 4′4-methylene were added and stirred for 20 min to prepare the superhydrophobic suspension. At room temperature, the suspension was sprayed on the substrates with a nozzle diameter of 0.6 mm at a spraying distance of 40 cm and air pressure of 0.6 bar, and then the coating was solidified in an 80 °C oven for 2 h to obtain a superhydrophobic coating.

### 3.4. Characterization

Fourier-transform infrared (FTIR) analysis was conducted on the lignin and PFDTES-Lignin samples by a FTIR spectrophotometer (Bruker VERTEX70, Rheinstetten, Germany). Each spectrum was recorded with 16 scans in transmittance mode with a resolution of 8 cm^−1^ within the range of 400–4000 cm^−1^. The chemical component analysis of lignin and modified lignin surface were examined and analyzed with an X-ray photoelectron spectrometer (XPS, ESCALAB Xi+, Waltham, MA, USA). Thermal analyses of the lignin and PFDTES-Lignin samples were performed using a thermogravimetric analyzer (TGA Q50, New Castle, DE, USA). Samples of about 10 mg were used in this analysis and in nitrogen atmosphere, the temperature increased from room temperature to 800 °C at a rate of 10 °C/min. A 400 MHz DRX-400 NMR instrument (Bruker, Germany) was used to test the 1H NMR spectra of the samples. The Folin-Ciocalteu (FC) method [48] was used to determine the phenolic hydroxyl content of the sample. The morphologies of lignin, PFDTES-Lignin, lignin coating and PFDTES-Lignin coating were observed by field emission scanning electron microscope (FE-SEM, Regulus8220, Hitachi, Japan). The interior structure of lignin coating and PFDTES-Lignin coating were observed by transmission electron microscope (TEM, JEOL-2100, Hokkaido, Japan). The article size and Zeta potential of lignin before and after modification is measured by Zeta potential and laser particle size analyzer (Nano ZS90, Malvern, UK). The surface morphology of lignin and PFDTES-lignin coatings were observed by atomic force microscopy (AFM, Multimode8, Karlsruhe, Germany) at a scanning rate of 256 Hz and scanning size of 10 μm.

## 4. Conclusions

In summary, we synthesized a superhydrophobic PFDTES-Lignin polymer via substitution reaction, which can be applied to glass, metal, paper and other substrates by spraying to prepare multifunctional superhydrophobic coatings. As a crosslinking agent, epoxy resin plays a role of crosslinking and adhesion, which can enhance the mechanical properties of coatings. The prepared PFDTES-Lignin coating has great repellency to water with contact angle of 164.7°. This composite coating not only puts up a good self-cleaning property, but also reserves its superhydrophobic property after 30 cycles of sandpaper wear with 100 g weight. Besides, the superhydrophobicity can be still retained after being stayed in 2 mol/L HCl, 0.25 mol/L NaOH and 2 mol/L NaCl solution for 30 min. The prepared PFDTES-Lignin polymer can be applied widely as superhydrophobic coating composite due to its fairly low-cost and scalable fabrications.

## Data Availability

All data generated or analysed during this study are included in this published article.

## Sample Availability

Samples of the compounds of PFDTES-Lignin are available from the authors.
